# Targeted Lipid Profiling Discovers Plasma Biomarkers of Acute Brain Injury

**DOI:** 10.1371/journal.pone.0129735

**Published:** 2015-06-15

**Authors:** Sunil A. Sheth, Anthony T. Iavarone, David S. Liebeskind, Seok Joon Won, Raymond A. Swanson

**Affiliations:** 1 Comprehensive Stroke Center and Department of Neurology, University of California Los Angeles, Los Angeles, California, United States of America; 2 QB3/Chemistry Mass Spectrometry Facility, University of California, Berkeley, California, United States of America; 3 Neurology and Rehabilitation Service, San Francisco Veterans Affairs Medical Center and Department of Neurology, University of California San Francisco, San Francisco, California, United States of America; University of South Florida, UNITED STATES

## Abstract

Prior efforts to identify a blood biomarker of brain injury have relied almost exclusively on proteins; however their low levels at early time points and poor correlation with injury severity have been limiting. Lipids, on the other hand, are the most abundant molecules in the brain and readily cross the blood-brain barrier. We previously showed that certain sphingolipid (SL) species are highly specific to the brain. Here we examined the feasibility of using SLs as biomarkers for acute brain injury. A rat model of traumatic brain injury (TBI) and a mouse model of stroke were used to identify candidate SL species though our mass-spectrometry based lipid profiling approach. Plasma samples collected after TBI in the rat showed large increases in many circulating SLs following injury, and larger lesions produced proportionately larger increases. Plasma samples collected 24 hours after stroke in mice similarly revealed a large increase in many SLs. We constructed an SL score (sum of the two SL species showing the largest relative increases in the mouse stroke model) and then evaluated the diagnostic value of this score on a small sample of patients (n = 14) who presented with acute stroke symptoms. Patients with true stroke had significantly higher SL scores than patients found to have non-stroke causes of their symptoms. The SL score correlated with the volume of ischemic brain tissue. These results demonstrate the feasibility of using lipid biomarkers to diagnose brain injury. Future studies will be needed to further characterize the diagnostic utility of this approach and to transition to an assay method applicable to clinical settings.

## Introduction

The detection of tissue-specific molecules plays a vital role in the diagnosis, management, and treatment of acute organ injury; however, such a marker of brain injury remains elusive. Imaging studies are frequently inconclusive in neurodegenerative conditions such as Alzheimer’s and Parkinson’s disease, and often cannot discriminate between pre-existing lesions and acute or ongoing processes. In emergency care settings where conditions such as acute ischemic stroke (AIS) and traumatic brain injury (TBI) are suspected, advanced imaging studies provide additional detail but can slow management decisions and are sometimes unavailable.

In response to these clinical needs, several brain-specific proteins have been examined as candidate plasma biomarkers of brain injury. To date, none have proven sufficiently sensitive or specific for routine clinical use[1–3]. For AIS, the ideal biomarker for this disorder would be readily detectible within 3–4.5 hours of symptom onset, the window for intravenous thrombolysis[4,5]. As a result, the clinical utility of the most commonly assayed protein markers, including S100b and neuron specific enolase (NSE), has been limited as neither rise in the plasma before 10–18 hours, nor do they correlate with infarct volume prior to 24 hours after injury[6,7]. Copeptin, a more recently evaluated molecule, has been shown to confer additional prognostic value when measured at the time of presentation, but is highly non-specific for the brain and may serve as a more general marker of physiologic stress[8,9]. Others are not detectable in the plasma earlier than 24 hours after the injury, exhibit small absolute increases, correlate poorly with injury severity, or are elevated in plasma by conditions other than brain injury [1,6,7,10–12].

To overcome these limitations, we evaluated the most abundant molecules in the brain, lipids. Lipids cross the blood-brain barrier more readily than proteins, and alterations in their brain levels develop early after injury[13]. In recent years, the detection and quantification of lipids has improved tremendously through techniques such as soft-ionization mass spectrometry[14]. These newer approaches focus on specific lipid subsets and thereby permit a previously unprecedented depth of analysis[15].

We targeted our lipid profiling to sphingolipids (SL), as this class of lipids is far more abundant in brain than in plasma. SLs account for about 22% of the dry weight of human adult white matter, but only 5% of plasma dry weight[16,17]. SLs can be further subdivided into a number of subclasses, which include ceramides (Cer), ceramide phosphates, glucosylceramides, sphingoid bases, and sphingomyelins (SM). Lipids in this manuscript were named by the sphingolipid subclass abbreviation (i.e. SM, Cer, etc.) followed by the number of carbons in the fatty acyl chains and then number of double-bonds, separated by a colon, consistent with prior studies[18,19]. For example “SM 37:1” refers to a sphingomyelin molecule with 37 carbons in the fatty acyl chains and 1 double bond.

By performing lipid profiling on 18 different mouse tissues we recently demonstrated that certain SL species are highly enriched in brain and have relatively low abundance in all other tissues[19]. Specifically, there is a 20–90 fold higher concentration of the sphingomyelin species SM 36:1 and 36:2 in the brain than in plasma. In this study we sought to determine whether these favorable biochemical features of SLs would allow for their early detection in circulating plasma after cerebral injury, in a manner that corresponds with the degree of injury. We evaluated changes in plasma SL concentrations in rodent models of AIS and TBI, and validated the feasibility of this approach in a small cohort of human patients presenting with AIS.

## Materials and Methods

### Ethics Statement

The animal studies were approved by the San Francisco Veterans Affairs Medical Center animal studies committee and performed in accordance with the National Institutes of Health Guide for the Care and Use of Laboratory Animals. IACUC approval was obtained prior to the initiation of the animal studies. In addition, ARRIVE guidelines have been followed for the animal work described in the manuscript[20]. Research involving human subjects was approved by the Institutional Review Board of the University of California, Los Angeles (IRB # 12–001740) and was conducted in compliance with the Health Information Portability and Accountability Act. A board-certified Neurologist interviewed all patients to determine the capacity of each individual participant to provide informed consent. If in the opinion of the physician the patient suffered from a compromised ability to provide consent, a surrogate consent procedure was instituted whereby the next of kin or legally authorized representative was given the opportunity to consent on the patient’s behalf. Formal written consent was obtained for all participants prior to the collection of blood samples.

### Study Design

This discovery-phase study was prospectively designed with a three-step approach of assay development, target identification and validation, with the purpose of investigating the utility of sphingolipid molecules as markers of acute brain injury. The first phase, assay development, established the ability of the lipid extraction and detection techniques to clearly differentiate sphingolipid content in brain tissue versus plasma. Two rodent species (rat and mouse) were used to ensure robustness of the assay. The second phase, SL target identification, analyzed sphingolipid content of plasma samples from mice after AIS and rats after TBI. Two types of injury models in two different species were used to ensure the robustness of the findings and improve generalizability. The third, validation phase evaluated the most promising SL species identified in mouse AIS in a small cohort of human AIS patients, using non-stroke patients presenting with similar stroke mimic symptoms as the control group.

### Brain Tissue Collection for SL analyses

Rats and mice were anesthetized with 2% isoflurane in 70% N_2_O/30% O_2_ using a facemask and perfused with chilled phosphate buffered saline to remove circulating blood. Brains were removed and kept at 4°C. A 100 mg wet-weight sample was taken from forebrain (the area injured in the AIS and TBI studies) and homogenized using a glass dounce 1 mL tissue grinder in phosphate buffered saline at 4°C. The homogenate was aliquoted, snap frozen on dry ice, and stored at -80°C until ready for extraction.

### Transient focal cerebral ischemia

Adult mice were fasted overnight with free access to water prior to surgery. Mice were then anesthetized with 2% isoflurane in 70% N_2_O/30% O_2_ using a facemask. Rectal temperature was maintained at 37 ± 0.5°C by using a homeothermic blanket throughout the surgical procedure. Regional cerebral blood flow was measured in all stroke animals using laser Doppler flowmetry (Moor Instruments, Wilmington, DE). Focal cerebral ischemia was induced by introducing a silicone rubber-coated nylon 6–0 monofilament (Doccol Corporation, Albuquerque, NM) into the right middle cerebral artery (MCA) for 45 minutes as described previously[21,22]. The right common carotid artery was exposed through a midline incision, separated from the vagus nerve and ligated. The external carotid artery was ligated. A filament was inserted through the external carotid artery stump and advanced along the internal carotid artery until the tip occluded the proximal stem of middle cerebral artery (MCA). Blood flow was restored by removal of the filament after 45 minutes. The wound was sutured, and the animal was moved to recovery chamber.

Blood was collected from the mice by terminal cardiac puncture into a heparinized syringe. At indicated time points, mice were anesthetized with 2% isoflurane in 70% N_2_O/30% O_2_ using a facemask. Rectal temperature was maintained at 37 ± 0.5°C by using a homeothermic blanket. Blood samples were kept on ice at 4°C and then centrifuged at 13,000 x *g* for five minutes at 4°C before being aliquoted into freezer vials, snap frozen on dry ice, and stored at -80°C until lipid extraction. At least 3 mice were used per time point. Control animals did not undergo sham surgery but did undergo the blood collection process.

Infarct volume was determined by staining with 2,3,5-triphenyltetrazolium chloride (TTC). At the time of euthanasia, mice were perfused with chilled phosphate buffered saline, brains were removed, and 2 mm coronal slices were prepared spanning the entire rostral-cuadal extent of the lesioned areas. The slices were incubated in 2% TTC in normal saline at 37°C for 15 minutes and then imaged. Infarct volume was calculated using the indirect method as previously described[23].

### Traumatic Brain Injury

A controlled cortical impact device (Hatteras Instruments, Cary, NC) was used to produce a unilateral TBI with varying injury volumes. Rats were fasted the night prior to surgery with free access to water, and anesthetized with intraperitoneal injections of ketamine (80 mg/kg) plus xylazine (8 mg/kg). Rectal temperature was maintained at 37 ± 0.5°C with a homeothermic blanket throughout the surgical procedure. The rats were placed in a stereotaxic frame with heads positioned to target the impact 3.0 mm left of the bregma. A midline scalp incision was made and a circular craniotomy was made while maintaining integrity of the dura. A controlled cortical impact device (Hatteras Instruments, Cary, NC) was used to produce a unilateral TBI with varying injury volumes[24]. For milder TBI, a 2.5 mm diameter impactor was used and programmed to 1.5 m/s velocity, 2.5 mm penetration depth, and 120 ms dwell time. For more severe TBI, a 5.0 mm impactor was used and the penetration depth was increased to 5 mm. The skin overlaying the site of injury was then closed and the animals were placed in a warmed recovery chamber until awake and ambulatory.

Prior to TBI a 26-gauge heparinized polyvinyl catheter (1 cm length) was introduced into femoral artery for blood sampling, and the skin sutured closed to completely internalize the catheter. At designated time points, rats were anesthetized with 2% isoflurane in 70% N_2_O/30% O_2_. The catheter was exposed, and blood was collected and handled as described for the samples collected from mice. The animals were then placed in a warmed recovery chamber. At least 3 rats were used per time point and per controlled cortical impact condition (severe and mild). Control animals did not undergo sham surgery but did undergo femoral artery catheter placement for blood collection. TBI lesion volume was determined by NeuN staining for viable neurons[24]. Rats were euthanized 1 or 7 days after TBI and perfused with 0.9% NaCl followed by 4% formaldehyde. Brains were removed and immersed in 4% formaldehyde for 24 hours followed by cryopotection in 20% sucrose. Coronal brain sections (thickness of 40 μm) were washed with 0.1 M phosphate buffer (PB) and incubated with blocking buffer (0.3% Triton X-100, 2% goat serum, and 0.1% bovine serum albumin in 0.1 M PB) at room temperature for 30 min. The sections were incubated with anti-NeuN (Millipore Co., Billerica, MA; 1:1000) overnight at 4°C. After washing, the sections were incubated with goat biotinylated goat anti-mouse IgG antibody (Amersham) at a dilution of 1:200 for two hours at room temperature. Serial coronal sections (480 μm) were photographed and the area devoid of NeuN staining on each section was calculated using Image J (National Institutes of Health, Bethesda, MD).

### Acute Stroke Patient Evaluation

All participants were patients presenting to the Ronald Reagan UCLA Emergency Department with symptoms concerning for acute stroke. Patients were included in this study if they had onset of stroke symptoms within 8 hours of presentation (or within 2 hours of presentation if symptoms were present upon awakening from sleep); were greater than 18 years of age; and were able to offer informed consent or had a suitable surrogate individual who could consent on their behalf. Enrollment was terminated once 14 patients were enrolled, based on sample size calculations (see below). The final clinical diagnosis was based on the results of a detailed evaluation of each patient by the Neurovascular Neurology service.

Blood samples were collected by peripheral vein venipuncture into heparin-containing tubes. Samples were kept on ice and then centrifuged immediately at 13,000xg for five minutes at 4°C. The plasma was collected and aliquoted into freezer vials for storage at -80°C. Lipid extraction and detection were performed in an identical manner to the animal studies.

Infarct size was determined by magnetic resonance imaging (MRI) performed at the time of patient presentation. Infarcted tissue was identified by a standard diffusion-weighted imaging protocol and quantified with a computer-assisted volumetric analysis program (Olea Medical, La Ciotat, France)[25]. In 2 stroke patients (out of 9), MRI imaging could not be obtained due to contraindication for MRI (implantable pacemaker) and as such these patients excluded from the imaging evaluation portion of this study.

### Sample Size Considerations

The plasma concentration of the SLs at the 24-hour time point in the mouse stroke model varied with a coefficient of variation of approximately 30% (see Results section). Assuming a 3-fold difference in SL levels between the stroke and stroke mimic patients, we calculated a greater than 80% power to detect this difference at an overall significance level of 5% (after Bonferroni correction for 30 different sphingolipid species) using 8 subjects in the stroke group and 5 subjects in the stroke mimic group.

### Sphingolipid Score

A sphingolipid score for human stroke evaluation was calculated by adding the median normalized (sphingolipid concentration divided by the median of the sphingolipid concentration of the stroke mimic patients) values of the two species with the greatest fold change in the mouse stroke model (SM 36:0 and Cer 42:1).

### Sphingolipid Extraction

All reagents were HPLC-grade unless otherwise noted, and all sample containers and pipette tips were glass or Teflon based. The method for the SL extraction is modified from a previously described method[17,26,27].

Twenty microliters of plasma (or 100 μL of diluted brain extract) were placed in 13 × 100 mm screw-capped borosilicate glass test tubes with Teflon caps (Fisher Scientific Catalog Number 14-933A, New Jersey, USA). 0.5 mL of methanol followed by 0.25 mL of chloroform (Fisher Scientific, New Jersey, USA) and 10 μL of internal standards (Sph/Cer Mixture I, Catolog Number LM-6002, Avanti Polar Lipids, Alabaster, AL) were added. Samples were then sonicated in a bath-type sonicator until they appeared evenly dispersed and then incubated 2 hours at 48°C in a heating block. Tubes were then cooled to room temperature and 75μL of 1M KOH was added. This mixture was sonicated briefly and then incubated for 30 minutes at 37°C. Samples were then cooled to room temperature and neutralized by addition of 16 μL of glacial acetic acid (Fisher Scientific, New Jersey, USA). pH was checked with test strips to verify near return to neutral pH 7.0. 1 mL of chloroform and 2 mL of water were added to each tubes. The solution was mixed gently and centrifuged at 300 x *g* for 5 minutes to separate the phases.

A Pasteur pipette was rinsed with chloroform and then used to remove the lower layer into another glass test tube, and the solvent was removed using a SpeedVac-type concentrator. The lipid residue was then redissolved in 75 μL methanol. The upper phase was re-extracted by adding 1 mL chloroform, mixing gently, and centrifuging as above. The lower layer was again transferred to another glass test tube using a Pasteur pipette that had been rinsed with chloroform, and the solvent was removed using a SpeedVac-type concentrator. The lipid residue was redissolved in 75 μL methanol. These two extracts were then pooled for a total of 150 μL, vortexed, and then centrifuged at 13,000x *g* to clarify. The supernatant was then transferred to an autosampler vial (Catalog number 225180, Wheaton, New Jersey, USA). Vials were stored at -80°C until ready for LC-MS analysis.

Protein content in mouse and rat brain and plasma was quantified using the Pierce BCA Protein Assay Kit (Thermo Scientific, Waltham, MA) according to the manufacturer’s directions. Lipid measurements in brain and plasma were normalized to protein content before comparison.

### Total sphingomyelin measurements

Total sphingomyelin content in plasma was measured using a biochemical assay (Sphingomyelin Colorimetric Assay Kit, Cayman Chemical, Ann Arbor, MI) per the manufacturer’s directions and normalized to plasma protein content.

### High performance liquid chromatography-mass spectrometry

Methanol (Optima grade, Fisher Scientific, New Jersey, USA), formic acid (99+%, Thermo Scientific Pierce, Waltham, MA), ammonium formate (99%, Alfa Aesar, Ward Hill, MA) and water purified to a resistivity of 18.2 MΩ cm (at 25°C) using a Milli-Q Gradient ultrapure water purification system (Millipore, Billerica, MA), were used to prepare mobile phase solvents for liquid chromatography-mass spectrometry.

Lipid extracts were analyzed using an Agilent 1200 liquid chromatograph (Agilent, Santa Clara, CA) that was connected in-line with an LTQ Orbitrap XL mass spectrometer equipped with an electrospray ionization source (ESI; Thermo Fisher Scientific, Waltham, MA). The LC was equipped with a C_4_ analytical column (Viva C4, 150 mm length × 1.0 mm inner diameter, 5 μm particles, 300 Å pores, Restek, Bellefonte, PA) and a 100 μL sample loop. Solvent A was 99.8% water/0.2% formic acid and solvent B was 99.8% methanol/0.2% formic acid (v/v). Solvents A and B both contained 5 mM ammonium formate. Lipid extract samples in autosampler vials sealed with septa caps were loaded into the autosampler compartment prior to analysis. The sample injection volume was 100 μL. The elution program consisted of isocratic flow at 30% B for 3 min, a linear gradient to 50% B over 0.5 min, a linear gradient to 100% B over 11.5 min, isocratic flow at 100% B for 5 min, a linear gradient to 30% B over 0.5 min, and isocratic flow at 30% B for 19.5 min, at a flow rate of 150 μL/min. The column and sample compartments were maintained at 40°C and 4°C, respectively. The injection needle was rinsed with a 1:1 methanol:water (v/v) solution after each injection to avoid cross-contamination between samples.

The column exit was connected to the ESI probe of the mass spectrometer using PEEK tubing (0.005” inner diameter × 1/16” outer diameter, Agilent, Santa Clara, CA). Mass spectra were acquired in the positive ion mode over the range *m*/*z* = 270 to 1150 using the Orbitrap mass analyzer, in profile format, with a mass resolution setting of 100,000 (at *m*/*z* = 400, measured at full width at half-maximum peak height). In the data-dependent mode, the five most intense ions exceeding an intensity threshold of 30,000 counts were selected from each full-scan mass spectrum for tandem mass spectrometry (MS/MS) analysis using collision-induced dissociation (CID) or pulsed-Q dissociation (PQD). MS/MS spectra were acquired in the positive ion mode using the linear ion trap, in centroid format, with the following parameters: isolation width 3 *m*/*z* units, normalized collision energy 30%, activation time 30 ms, activation Q 0.25 for CID, activation Q 0.50 for PQD, and default charge state 1+. A parent mass list was used to preferentially select ions of interest for MS/MS analysis. To avoid the occurrence of redundant MS/MS measurements, real-time dynamic exclusion was enabled to preclude re-selection of previously analyzed precursor ions, using the following parameters: repeat count 2, repeat duration 30 s, exclusion list size 500, exclusion duration 60 s, and exclusion width 0.1 *m*/*z* unit. Data were processed and analyzed using Xcalibur software (version 2.0.7 SP1, Thermo Scientific, Waltham, MA), mMass software (version 5.4.1), and LIPID MAPS Online Tools[28,29]. Sphingolipid identification was made by exact mass measurements coupled with MS/MS measurements, and searched against the LIPID MAPS Online Database. This database contains annotated entries for all known lipids as well as their modified products (i.e. oxidation, glycosylation, etc.). Sphingolipid species used in this analysis were confined to those present and characterized within this database.

### Statistical Methods

**Fig 1:**
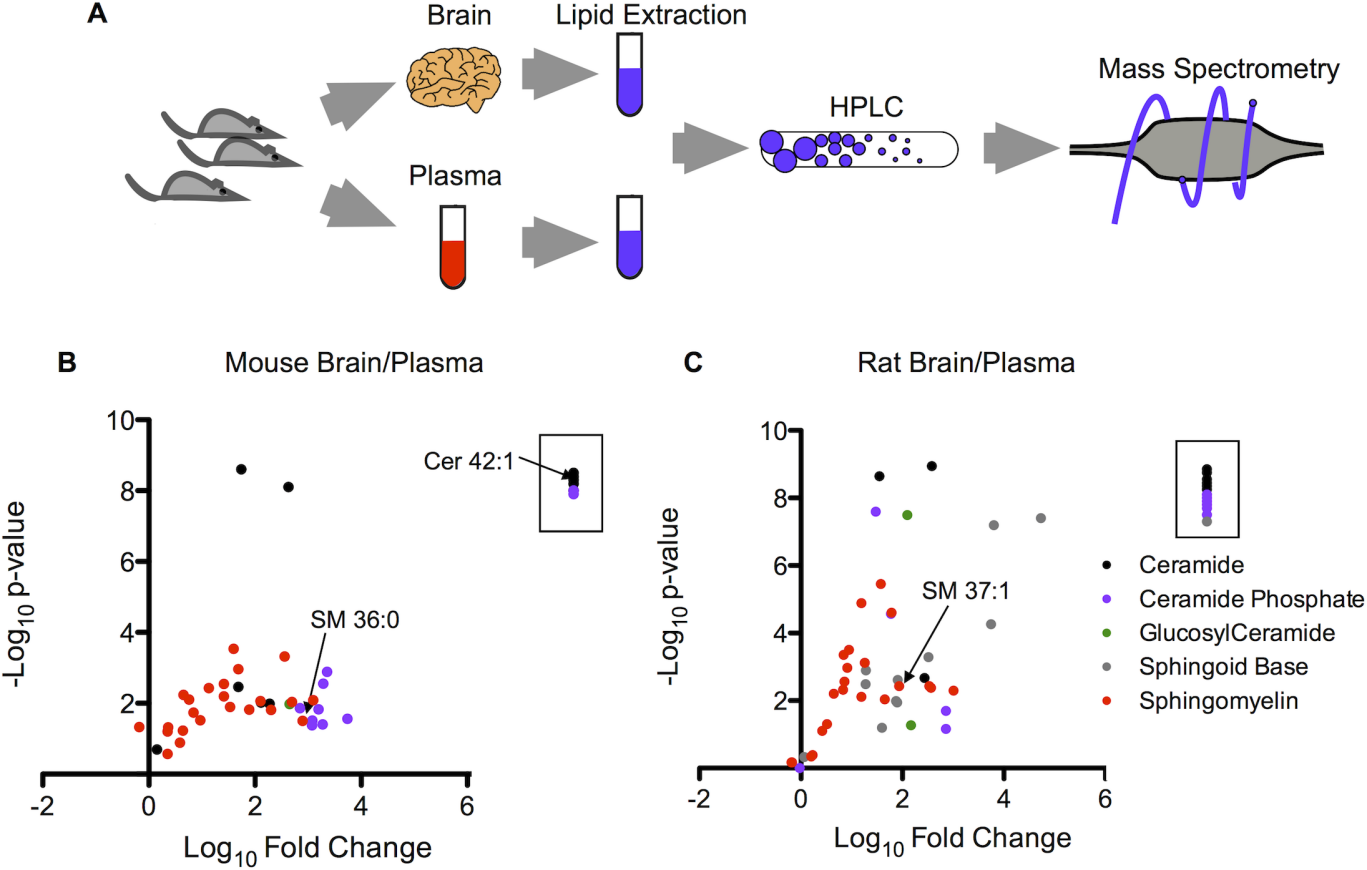
Targeted sphingolipid profiling in mouse and rat brain and plasma. **(a)** Schematic of the workflow for the isolation of rodent brain and plasma, lipid extraction, and HPLC MS/MS. **(b)** Volcano plot of the negative log_10_ of the p-value (Student’s t-test, two-tailed) versus the log_10_ of the normalized (to protein content) fold difference in concentration in brain over plasma for the 45 sphingolipids identified in mouse brain and plasma. Species within the box (8 total) were found in the brain but not identified in the plasma. *n* = 3 per sample. **(c)** Volcano plot of the negative log_10_ of the p-value (Student’s t-test, two-tailed) versus the log_10_ of the normalized fold difference in concentration in brain over plasma for the 56 sphingolipids identified in rat brain and plasma. Species within the box (13 total) were found in the brain but not identified in the plasma. *n* = 3 per sample.

**Fig 2:**
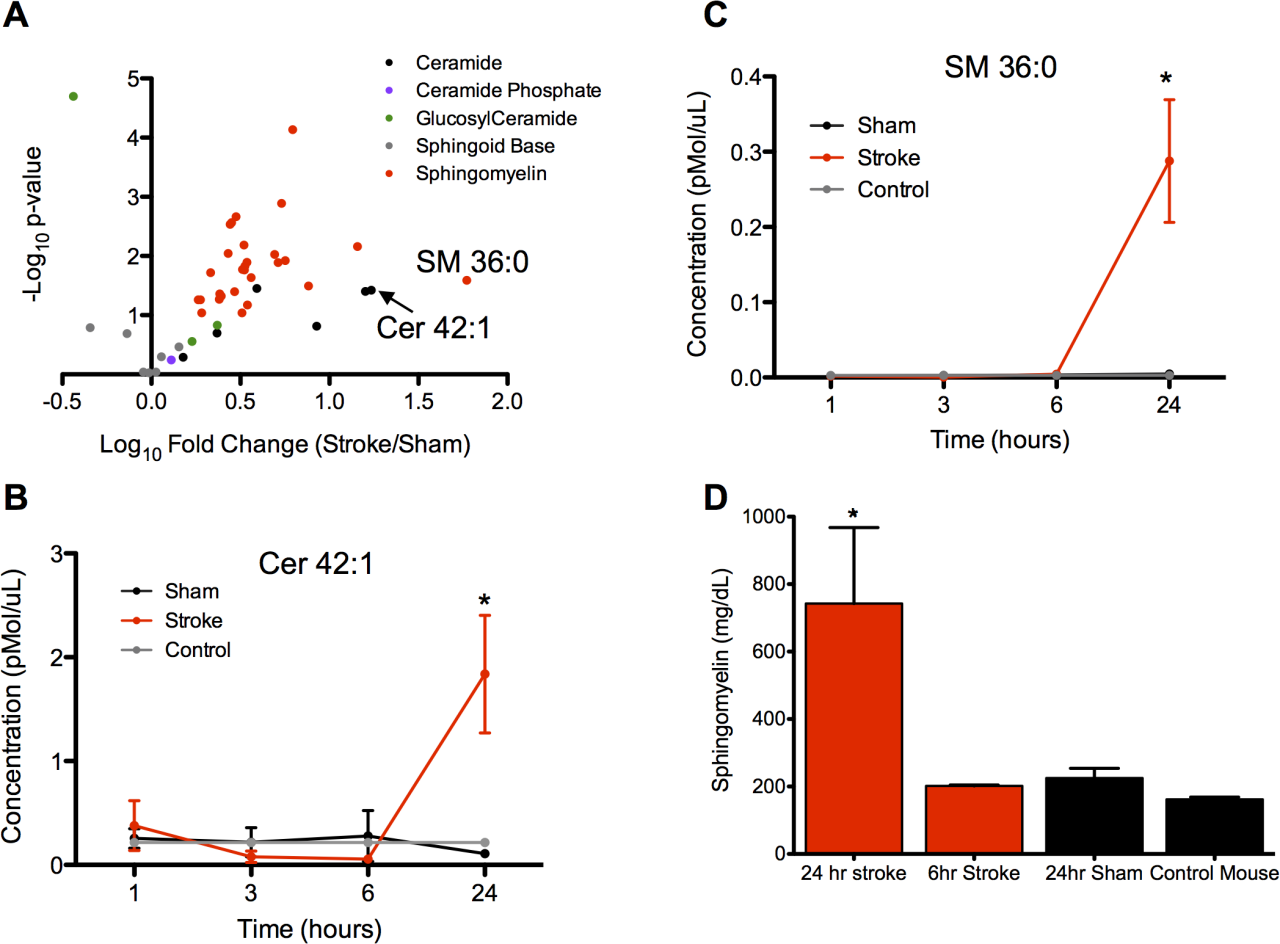
Sphingolipid profiling in middle cerebral artery occlusion in mice. **(a)** Volcano plot of the negative log_10_ of the p-value (Student’s t-test, two-tailed) versus the log_10_ of the normalized fold change in plasma concentration of stroke over sham animals at the 24 hour time point. Selected top performing sphingolipids are indicated by labels. *n* = 3 per sample. **(b** and **c)** Time course of the two sphingolipids with the greatest fold change. Red line indicates stroke animal, black line indicates sham animal, and grey line indicates control animal without any surgical procedure but with identical blood collection process. Data are shown as mean ± SEM. * indicates p<0.05, Student’s t-test, two-tailed. *n* = 3 per sample. **(d)** Total sphingomyelin concentration in plasma as measured by biochemical assay. * indicates p<0.05, one-way ANOVA with Dunnett’s multiple comparisons test.

**Fig 3:**
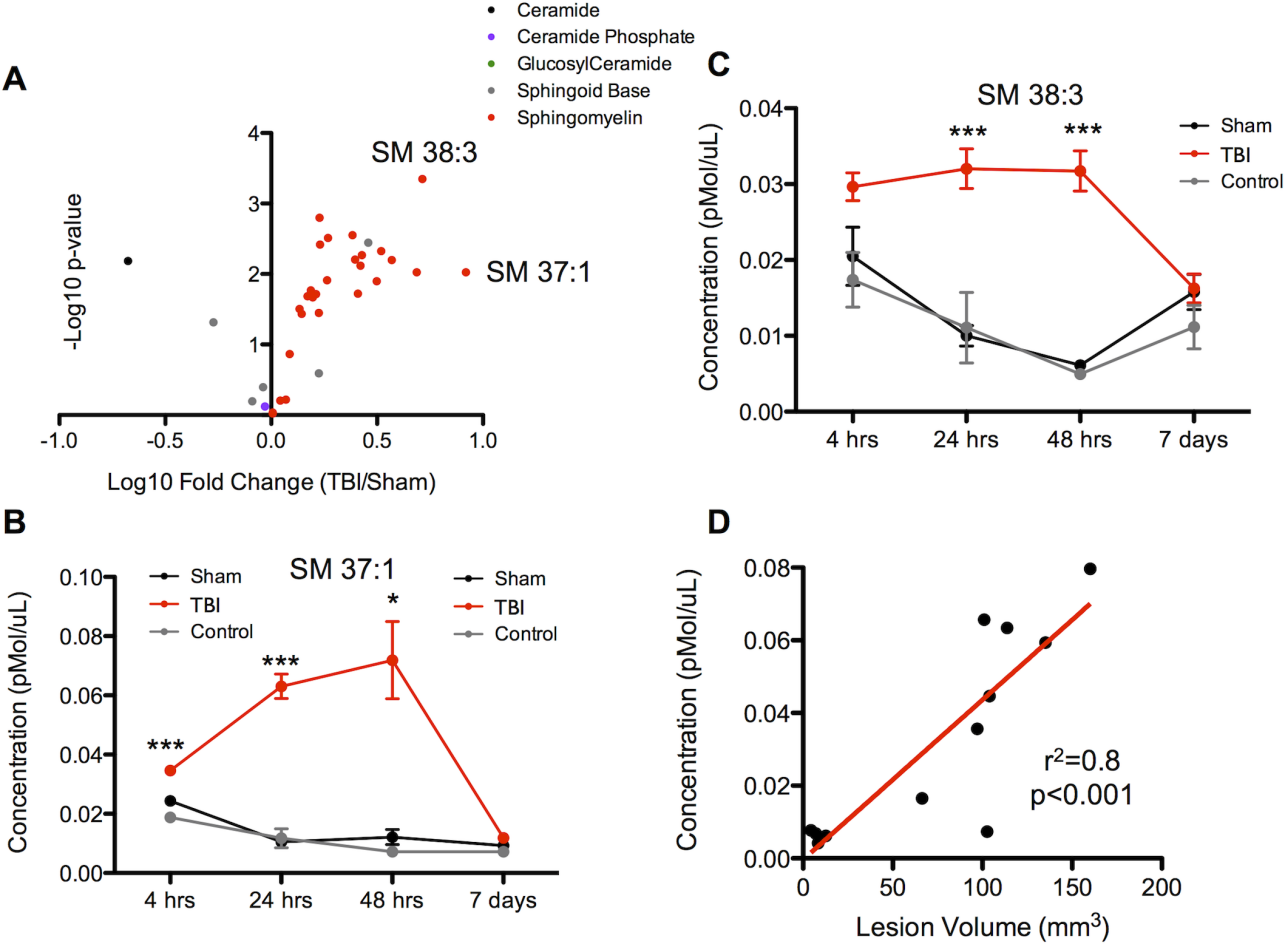
Sphingolipid profiling in controlled cortical impact in rats. **(a)** Volcano plot of the negative log_10_ of the p-value (Student’s t-test, two-tailed) versus the log_10_ of the normalized fold change in plasma concentration of TBI over sham animals at the 48 hour time point. Selected top performing sphingolipids are indicated by labels. *n* = 4 per sample. **(b** and **c)** Time course of the two sphingolipids with the greatest fold change. Red line indicates TBI animal, black line indicates sham animal, and grey line indicates control animal without any surgical procedure but with identical blood collection process. Data are shown as mean ± SEM. * indicates p<0.05, *** indicates p<0.001, Student’s t-test. *n* = 4 per sample. **(d)** Scatter plot demonstrating the relationship between the plasma concentration of the sphingolipid with the greatest fold change (SM 37:1) at 48 hours versus the volume of injured brain. Linear correlation shown in red (p<0.001, Pearson’s correlation coefficient).

All statistical analyses were performed using commercially available software (Prism 5.0a, GraphPad Software, La Jolla, CA). p<0.05 was considered statistically significant. Data in Figs 1–3 are displayed in volcano plot format, which graphs statistical significance of the data (in the form of p-value) against the fold change or fold difference. Species in the top right corner of the plot therefore are the most significant as well as with the largest fold difference. Continuous data were represented as mean ± SEM if the D’Agostino & Pearson omnibus normality test was passed. Otherwise, they were presented as median [IQR].

## Results

Our targeted lipid profiling approach (Fig 1A) provided a broad linear range for all SL subclasses, and the median coefficient of variation (CV) for technical replicates of identical plasma samples was 7% [IQR 5–12]. Using this technique, we identified many SLs that are highly abundant in the brain compared to plasma. Of the 45 SLs identified in mice and 56 SLs identified in rats (Fig 1B and 1C), nearly all were present at much higher concentration in the brain, many with a greater than 1000-fold difference. In addition, there were a number of species found in the brain (8 in the mouse and 13 in the rat) that were undetectable in the plasma. The majority of SL species detected were sphingomyelins (SM) although members of all SL subclasses were seen. Sphingoid bases, ceramide phosphates, and glucosyl ceramides were not as readily detected.

We then sought to determine changes in circulating plasma SL levels after ischemic stroke in the mouse. Plasma collected 24 hours after the injury revealed a dramatic increase in SL levels in the stroke compared to sham animals, with the majority of the top performing species being SM and Cer, with increases of up to 60 fold (Fig 2A). The top species were Cer 42:1 (1.8 vs. 0.1 pmol/μL, stroke vs. sham, p<0.05) and SM 36:0 (0.3 vs. 0.005 pmol/μL, stroke vs. sham, p<0.05). The rise in SL levels occurred between 6 and 24 hours after the injury, and this time course was highly consistent across the top ten lipids (Fig 2B and 2C and S1 Fig). Before this time point, SL levels in stroke animal plasma were comparable to those of the sham and control animals. The median CV for biological replicates in the 24 hour sham and stroke groups were 29% [IQR 15–51] and 31% [IQR 22–40] respectively. The changes in plasma SM after stroke were confirmed by measuring total SM levels (Fig 2D) using an independent biochemical assay.

Next, we determined the changes in plasma SL levels in a second brain injury model, TBI, in rats. We found a similar robust increase in circulating SL following injury (Fig 3A), with SMs being the most prominent species. The SMs with the largest magnitude increase were SM 37:1 and SM 38:3. The rise induced by TBI was smaller than in the murine stroke model, possibly reflecting the two-fold smaller volume of injury in the TBI compared to stroke, but the elevations were detectable earlier after TBI (Fig 3B and 3C). The time course of the rise and subsequent fall in plasma levels was similar for the top ten species with the greatest fold change (S2 Fig). The median CV for biological replicates in the 48-hour sham and TBI groups were 15% [IQR 8–21] and 23% [16–30] respectively. The increase in circulating SL levels in the early time points in all three groups (sham, control and TBI) may have resulted in part from the surgical placement of the intra-arterial catheter used for serial blood collection, but the animals that received CCI continued to have elevations in SL levels in excess of the sham and control animals. Further, by varying the degree of CCI injury, we found that the increase in SM concentration was directly proportional to the lesion volume (r^2^ = 0.8, p<0.05) (Fig 3D). For each individual SL species, the relative fold change after TBI compared to sham surgery correlated directly with the fold change for that species seen in the stroke model, as shown in S3 Fig.

We then sought to validate this technique in a patient population presenting with symptoms concerning for acute stroke. We enrolled patients (n = 14) who presented emergently to the hospital and collected plasma samples within 30 minutes of arrival. Of these patients, 9/14 (64%) were ultimately diagnosed with stroke, and 5/14 (36%) were diagnosed as having a stroke mimic. Clinical features of these patients are provided in S1 Table. Of the 9 patients diagnosed with stroke, 4/9 (44%) of patients underwent blood sampling in under 3 hours from when symptoms were first observed. Stroke mimic patients were ultimately diagnosed with seizure, complicated migraine, Bell’s palsy or factitious disorder. Consistent with prior studies, the stroke mimic patients tended to be younger and more often female [30].

**Fig 4:**
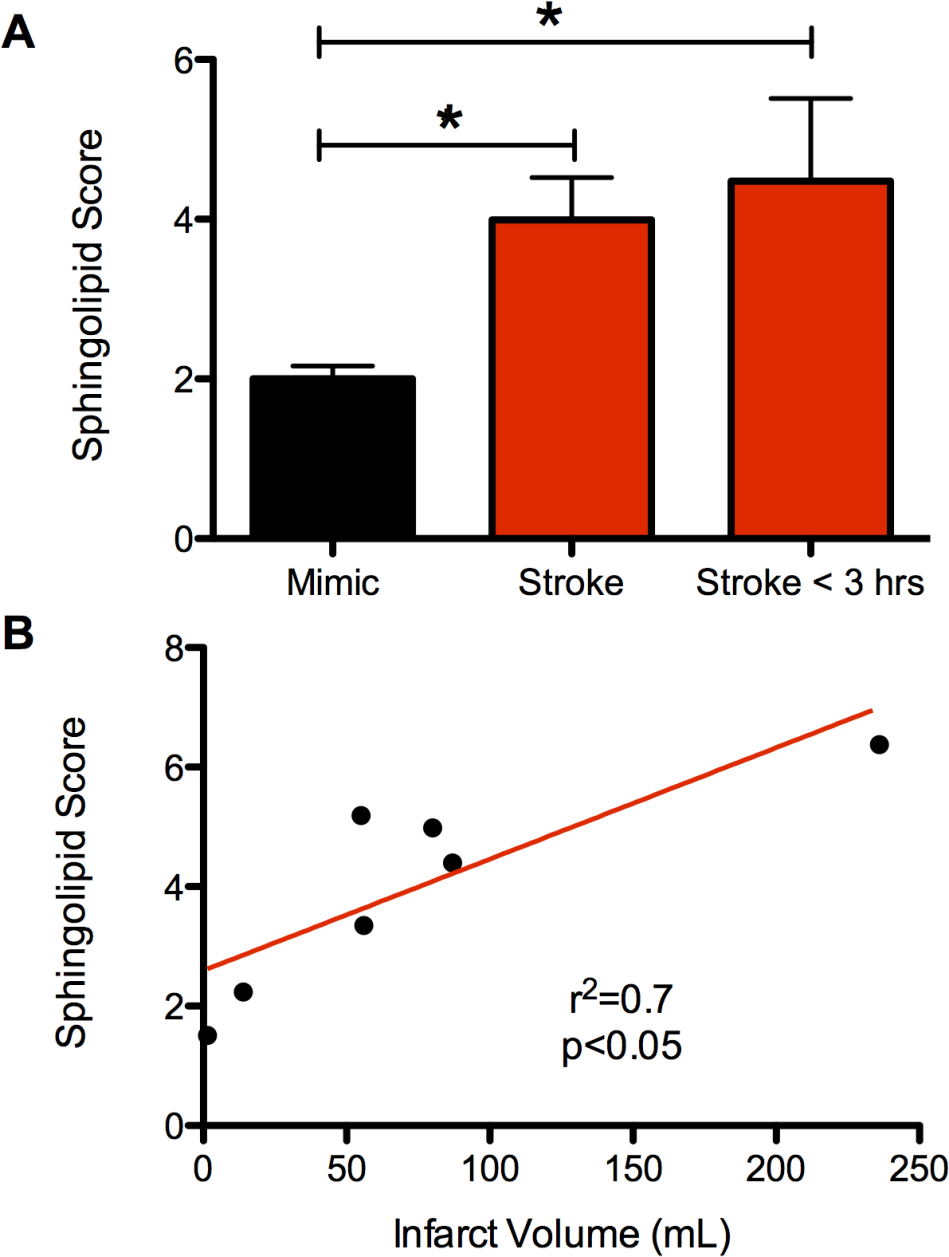
Sphingolipid profiling in patients presenting with acute neurological deficits concerning for stroke. **(a)** SL Score from plasma at the time of hospital arrival for patients ultimately diagnosed as stroke and those diagnosed as having a stroke mimic. Three groups were included in the comparison: all patients presenting with stroke, all patients presenting with stroke mimics, and the subset of stroke patients from whom blood draw was within 3 hours of when the symptoms were first observed. Data are shown as mean ± SEM. * indicates p<0.05, Student’s t-test, two-tailed. **(b)** Scatter plot demonstrating the relationship of the SL score with final infarct volume as determined by MRI diffusion weighted imaging. Linear correlation shown in red (p<0.05, Pearson’s correlation coefficient).

Building from the SL targets identified in the mouse stroke studies, we constructed an SL Score consisting of the normalized sum of the plasma concentration of the two species with the greatest fold change from this model (namely, SM 36:0 and Cer 42:1). By including one SM and one Cer, we reduced intra-subclass variability, while maintaining a statistically simple model with a numerical value that could still be understood intuitively. As shown in Fig 4A, patients ultimately diagnosed with stroke were found to have on average over two-fold higher SL scores than the stroke-mimic patients. The subset of stroke patients who presented and had blood drawn within three hours of when their symptoms were first observed also had greater SL scores than those diagnosed as stroke-mimic. The SL score also correlated with the volume of ischemic brain tissue (MRI diffusion-weighted imaging volume) in the stroke patients (Fig 4B). We then calculated receiver-operator curve (ROC) and area under the curve (AUC) statistics for the ability of the SL score to differentiate stroke from stroke mimic. This curve can be found on S4 Fig. The AUC was found to be 0.87.

## Discussion

By focusing on a class of lipids abundant in the brain, we have identified a subset of SLs which, in animal models, rise in the plasma after traumatic brain injury and stroke and correlate with the severity of injury. The two SLs with the most marked elevations were incorporated into an “SL score”. In a small cohort of patients presenting with symptoms of AIS, we showed that the SL species identified in the animal model rise at early time points and correlate with degree of injury. The SL score was increased by over two fold in plasma collected from stroke patients at time points of 3 hours or less from when symptoms were first noticed.

From our ROC curve for the SL score, we found an AUC of 0.87 to differentiate stroke versus stroke mimic on blood samples taken at the time of patient arrival to the hospital. Though our sample size is limited, this value is elevated when compared against protein biomarkers of stroke. An algorithm that combined the value of four protein markers (matrix metalloproteinase 9, D-dimer, S100b, and B-type natriuretic peptide) was found to have an AUC of 0.69 for differentiating stroke from mimic[30]. For differentiating patients with traumatic brain injury against those without traumatic brain injury using blood collected within 3 hours of hospital arrival, the AUC for S-100b was found to be 0.66, and for NSE, 0.67[31]. GFAP was shown to have an AUC of 0.79 to detect whether patients with TBI would have computerized tomography (CT) scan findings[32].

One explanation for SL presence in the blood at time points earlier than protein markers may be the increased blood-brain barrier permeability of small lipid-soluble molecules. Whereas larger, hydrophilic protein markers stay sequestered in cerebral tissue until the blood-brain barrier breaks down over several hours following the injury, lipids can escape earlier[33]. In addition, SLs within the brain are found at high concentrations in cell membranes and the myelin sheets of white matter oligodendroglia. Because of this localization, coupled with the rapid changes in oligodendrocyte membranes after injury[34], plasma SL levels may rise before cell death has occurs. Such a mechanism may also explain why SL levels rise earlier in human stroke than the mouse model, as human brains contain far more white matter per volume of brain[35]. As such, rodent injury models may greatly underestimate the sensitivity of this SL-based assay. Indeed, SM content is significantly higher in the human brain than rodent brain–approximately 15% of the lipid content of human brain and only 5.7% of the rat[36,37].

The SL targets that we identified in our pre-clinical rodent models consisted primarily of SM and Cer species. Both these classes of lipids are concentrated in cerebral white matter and in greater abundance in the brain compared to plasma[16,17]. Per unit dry weight, ceramides are in approximately 5-fold greater concentration in white matter compared to plasma. Specifically, we also found that the two species with the greatest fold change in the mouse stroke model that then constituted the SL score (SM 36:0 and Cer 42:1) were in very high abundance in the brain compared to plasma (56 and 788 fold increase in mouse brain compared to plasma).

A consideration in the development of lipid-based biomarkers of injury is plasma half-life. The major source of ceramide production is from sphingomyelinase-driven breakdown of sphingomyelin. Thus, it is conceivable that SM and Cer biomarkers of brain injury will demonstrate different plasma concentration time courses after brain injury. Moreover, a significant proportion of plasma SLs may be contained within circulating microvesicles such as exosomes. Exosomes have been shown to pass through the blood-brain-barrier and their structure may allow for longer half-life in plasma[38].

In this study we performed targeted lipid profiling and did not attempt to identify all possible brain or plasma lipids, though such efforts have been performed previously using multiple extraction and detection techniques[17]. Further studies will be needed to quantify the effect on plasma SL level of additional variables such as time after injury, region of brain involved, patient age, and others, with the goal of establishing a threshold level at which the presence or absence of acute cerebral injury can be confidently determined. Our finding that stroke mimic patients tend to be younger and female is consistent with prior studies, and the potential impact of these variables on our results will need to be studied in additional detail in a larger cohort[30]. Further, although in our prior work we demonstrated high specificity for sphingolipid species to brain tissue compared with other organs in the mouse, this specificity may not be the case in humans[19]. Alternate lipid sources, including bone marrow, adipose tissue, skin, and other sources could limit the ultimate specificity of this approach in detecting brain injury in settings where polytrauma is a consideration. Correlating the markers with clinically based outcome measures and neurological function scales will also be important. Finally, transitioning from a mass spectrometry based approach, which is not readily available in clinical settings, to a biochemical and ideally bedside means of identifying key SLs would aid in advancing the assay.

These results demonstrate the feasibility of lipid profiling in identifying circulating markers of acute brain injury. While we focus on the acute brain injury syndromes of AIS and TBI in this work, SLs may also provide biomarker identification for ongoing brain injury in neurodegenerative diseases such as Alzheimer’s disease, as well as multiple sclerosis and other chronic neurological disorders. These findings and approach represent a novel direction towards a clinically important need.

## Supporting Information

S1 Fig Plasma levels over time of the next top 8 sphingolipids with the greatest fold changes following middle cerebral artery occlusion in mouse. **(a-i)** Time courses for plasma concentration of the remaining top 2–10 sphingolipids in the mouse stroke model. A consistent pattern of minimal signal up to six hours followed by appearance in the plasma between 6 and 24 hours is seen across all 8 species. Red line indicates stroke animal, black line indicates sham animal, and grey line indicates control animal without any surgical procedure but with identical blood collection process. * indicates p<0.05, ** indicates p<0.01, *** indicates p<0.001, Student’s two-tailed t-test.
(TIFF)

S2 Fig Plasma levels over time of next top 8 sphingolipids with the greatest fold changes following controlled cortical impact in rat. **(a-i)** Time courses for plasma concentration of the remaining top 2–10 sphingolipids in the rat TBI model. A consistent pattern of low level but significant appearance in the plasma at 4 hours followed by rising levels peaking at 48 hours and a return to control levels at one week is seen in all 8 species. Red line indicates stroke animal, black line indicates sham animal, and grey line indicates control animal without any surgical procedure but with identical blood collection process. * indicates p<0.05, ** indicates p<0.01, *** indicates p<0.001, Student’s two-tailed t-test.
(TIFF)

S3 Fig Correlation of sphingolipid species in TBI and AIS models. Scatter plot demonstrating the relationship between the fold changes in plasma concentration of sphingolipid species after TBI in rat over sham versus stroke in mouse over sham. Linear correlation indicated by the red line (p<0.001, Pearson’s correlation coefficient). Dashed black line indicates 95% confidence intervals.
(TIFF)

S4 Fig Receiver-operator curve for the SL score to differentiate stroke from stroke mimic. Curve calculated from SL values from blood samples taken at the time of patient arrival to the ED. Area under the curve (AUC) value was calculated to be 0.87.
(TIFF)

S1 Table Patient Characteristics. (DOCX)
