# Supplementary figures and images for: Targeted Lipid Profiling Discovers Plasma Biomarkers of Acute Brain Injury
Source: PLoS One. 2015 Jun 15;10(6):e0129735. doi: 10.1371/journal.pone.0129735 (PMC4468135; doi:10.1371/journal.pone.0129735)

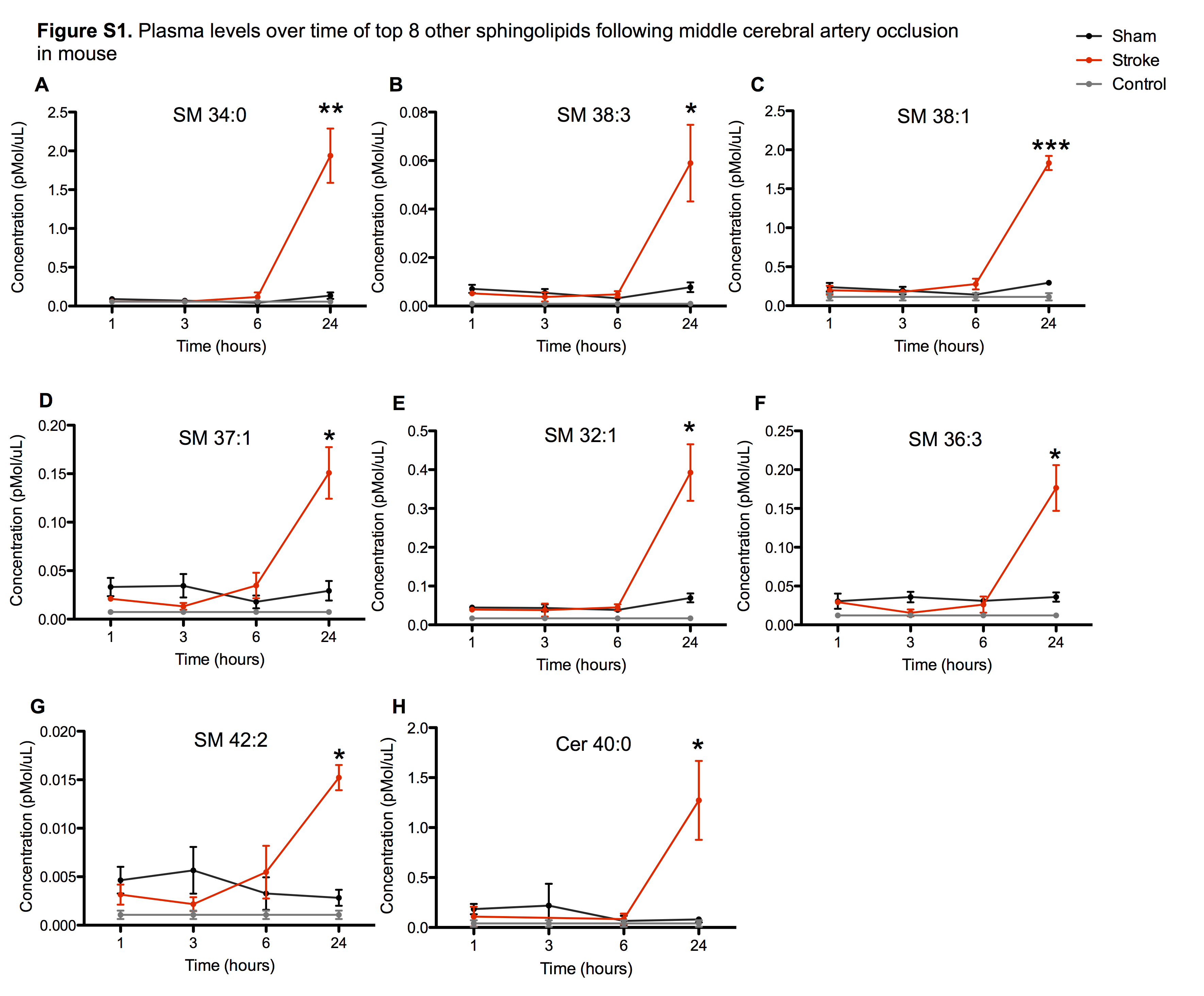

Supplement: S1 Fig — (a-i) Time courses for plasma concentration of the remaining top 2–10 sphingolipids in the mouse stroke model. A consistent pattern of minimal signal up to six hours followed by appearance in the plasma between 6 and 24 hours is seen across all 8 species. Red line indicates stroke animal, black line indicates sham animal, and grey line indicates control animal without any surgical procedure but with identical blood collection process. * indicates p<0.05, ** indicates p<0.01, *** indicates p<0.001, Student’s two-tailed t-test. (TIFF) [file pone.0129735.s001.tiff]

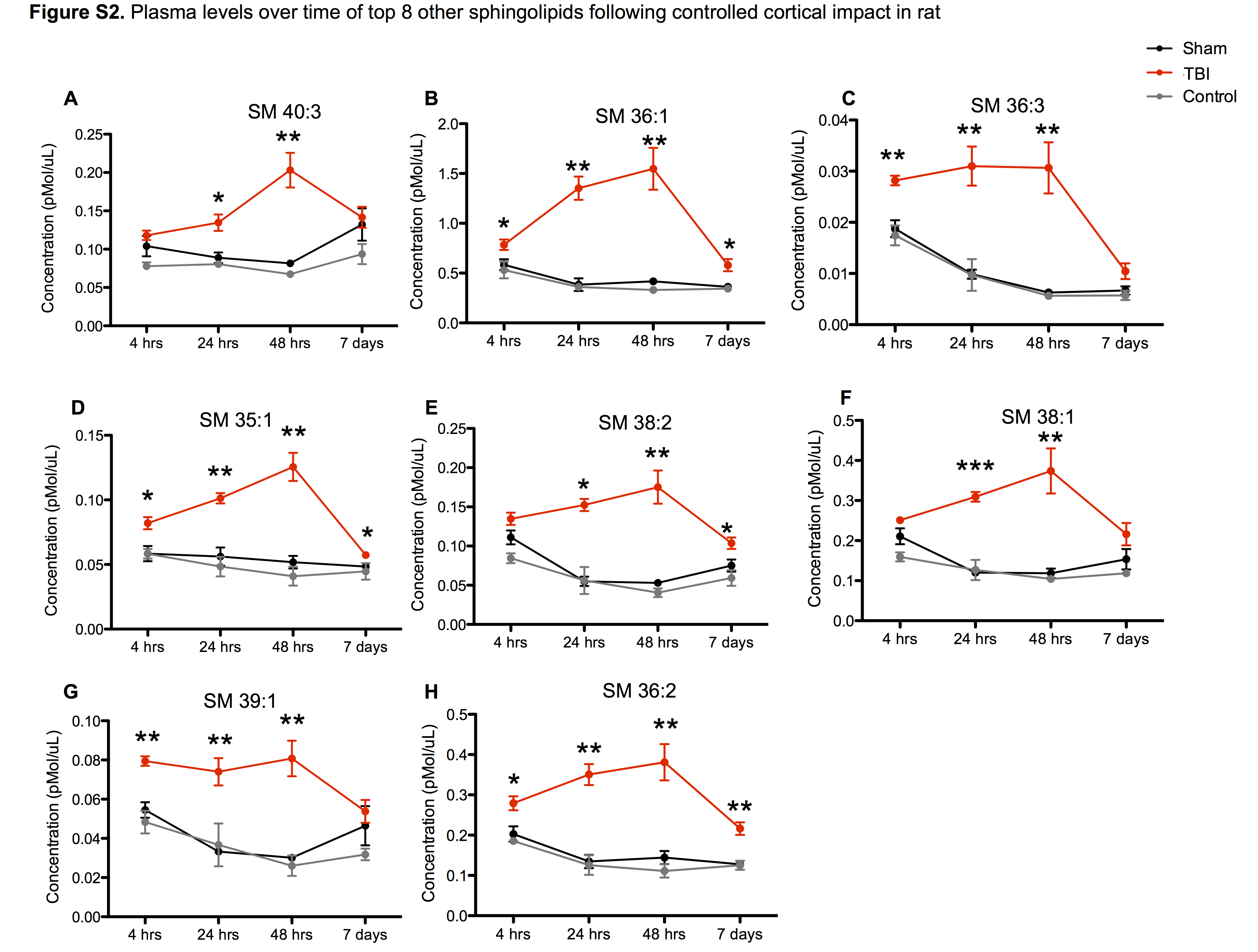

Supplement: S2 Fig — (a-i) Time courses for plasma concentration of the remaining top 2–10 sphingolipids in the rat TBI model. A consistent pattern of low level but significant appearance in the plasma at 4 hours followed by rising levels peaking at 48 hours and a return to control levels at one week is seen in all 8 species. Red line indicates stroke animal, black line indicates sham animal, and grey line indicates control animal without any surgical procedure but with identical blood collection process. * indicates p<0.05, ** indicates p<0.01, *** indicates p<0.001, Student’s two-tailed t-test. (TIFF) [file pone.0129735.s002.tiff]

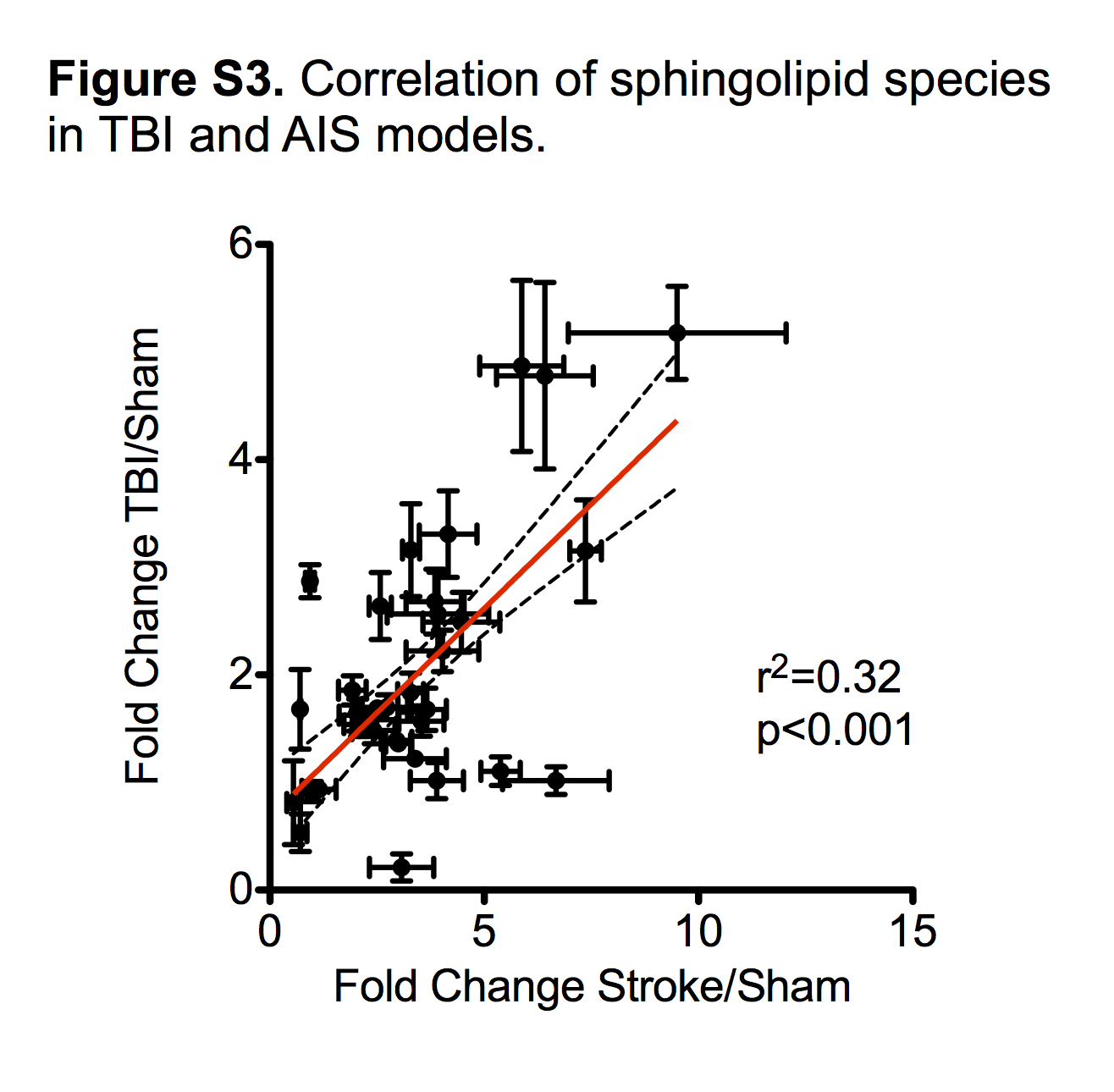

Supplement: S3 Fig — Scatter plot demonstrating the relationship between the fold changes in plasma concentration of sphingolipid species after TBI in rat over sham versus stroke in mouse over sham. Linear correlation indicated by the red line (p<0.001, Pearson’s correlation coefficient). Dashed black line indicates 95% confidence intervals. (TIFF) [file pone.0129735.s003.tiff]

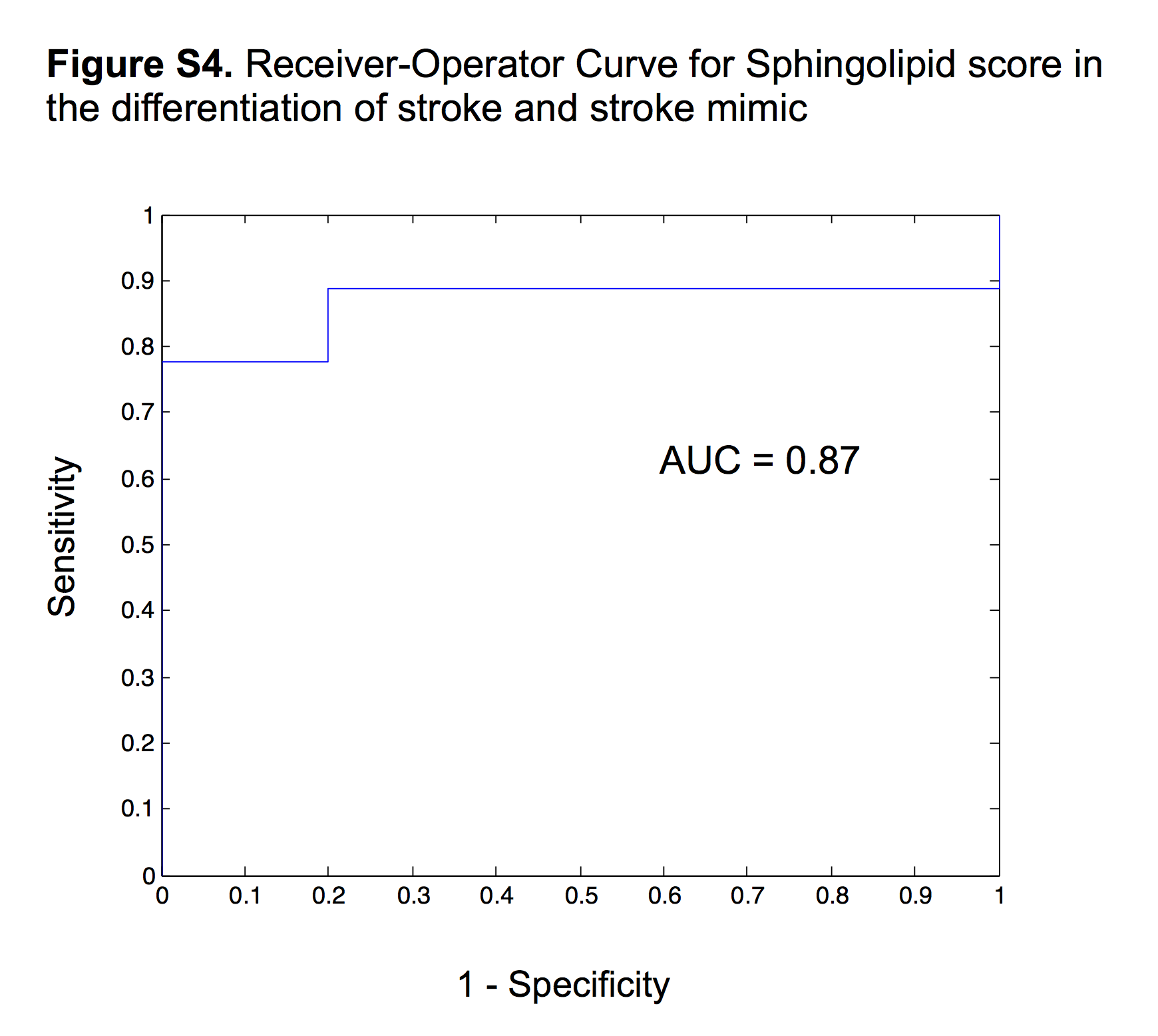

Supplement: S4 Fig — Curve calculated from SL values from blood samples taken at the time of patient arrival to the ED. Area under the curve (AUC) value was calculated to be 0.87. (TIFF) [file pone.0129735.s004.tiff]
