# Supplementary material for: Targeted Lipid Profiling Discovers Plasma Biomarkers of Acute Brain Injury
Source: PLoS One. 2015 Jun 15;10(6):e0129735. doi: 10.1371/journal.pone.0129735 (PMC4468135; doi:10.1371/journal.pone.0129735)
Supplement: S1 Table — (DOCX) [file pone.0129735.s005.docx]

**Supplementary Tables**

| **Table S1.** Patient Characteristics | |  |  |
| --- | --- | --- | --- |
|  | **Stroke** | **Stroke Mimic** | **p-value** |
| Number | 9 | 5 |  |
| Age | 85 [73-89] | 55 [46-65] | <0.01 |
| Female Gender | 3 (33%) | 5 (100%) | <0.05 |
| NIH Stroke Scale | 14 [3-22] | N/A | N/A |
| Time from last known well to blood draw (min) | 435 [120-780] | 240 [90-540] | 0.27 |
| Time from symptoms first observed to blood draw (min) | 388 [80-660] | 240 [90-540] | 0.29 |
| Infarct volume on MRI (mL)* | 60 [1.5 - 236] | 0 | N/A |
| Treated with tPA | 4 (44%) | 0 (0%) | N/A |
| Final Diagnosis |  |  | N/A |
| - MCA occlusion | 7 (78%) | N/A |  |
| - Lacunar Stroke | 1 (11%) | N/A |  |
| - Basilar artery occlusion | 1 (11%) | N/A |  |
| - Complicated Migraine | N/A | 2 (40%) |  |
| - Seizure | N/A | 1 (20%) |  |
| - Factitious Disorder | N/A | 1 (20%) |  |
| - Bell's Palsy | N/A | 1 (20%) |  |
| Discharge Destination |  |  | <0.01 |
| - Home | 1 (11%) | 5 (100%) |  |
| - Skilled Nursing Facility | 5 (56%) | 0 (0%) |  |
| - Death | 3 (33%) | 0 (0%) |  |
|  |  |  |  |
| Note: All data presented as median [range] or number (%) unless otherwise specified. | | |  |
| * Two patients were unable to undergo MRI due to the presence of an implantable pacemaker,  and as such accurate measurements of final infarct volume could not be made. | | |  |
